# Lymphovenous Bypass as an Adjunct to Standard Care for Diabetic Peripheral Neuropathy: Protocol for a Randomized Assessor-Blinded Superiority Trial

**DOI:** 10.2196/84826

**Published:** 2026-06-08

**Authors:** Hsin-Ying Lee, Chih-Yuan Wang, Tien-Jyun Chang, Chi-Chao Chao, Sung-Tsang Hsieh, Nai-Chen Cheng, Sung-Chuan Chao

**Affiliations:** 1 Department of Surgery, National Taiwan University Hospital, College of Medicine, National Taiwan University, Taipei, Taiwan; 2 Department of Internal Medicine, National Taiwan University Hospital, College of Medicine, National Taiwan University, Taipei, Taiwan; 3 Department of Neurology, National Taiwan University Hospital, College of Medicine, National Taiwan University, Taipei, Taiwan; 4 Department of Traumatology, National Taiwan University Hospital, College of Medicine, National Taiwan University, No. 7, Chung-Shan South Road, Taipei, 100225, Taiwan, 886 2-23123456, 886 2-23825540

**Keywords:** diabetic peripheral neuropathy, diabetic foot ulcer, lymphovenous bypass, diabetic peripheral neuropathy pain, Sudoscan

## Abstract

**Background:**

Diabetic peripheral neuropathy (DPN) is a length-dependent, symmetric sensorimotor polyneuropathy with a substantial global and regional burden. Current pharmacologic options are largely symptomatic and do not modify the disease. Lymphovenous bypass (LVB), a supermicrosurgical procedure established for lymphedema, may modulate lymphatic-immune-microvascular dysfunction relevant to DPN.

**Objective:**

The primary objective is to determine whether LVB combined with standard of care (SOC) improves small-fiber and autonomic function compared with SOC alone at 6 months. Secondary objectives are to evaluate the effects of LVB on large-fiber function, neuropathic pain, ulcer healing, quality of life, and relevant biomarkers, as well as to characterize the safety profile of LVB.

**Methods:**

This is a SPIRIT (Standard Protocol Items: Recommendations for Interventional Trials)–aligned, single-center, randomized, controlled, parallel-group superiority trial with a 2:1 allocation (LVB+SOC vs SOC alone). Randomization is stratified based on the presence or absence of active diabetic foot ulcers, as defined by the International Working Group on the Diabetic Foot and Infectious Diseases Society of America criteria. In total, 60 adults aged 20 to 80 years with confirmed DPN will be enrolled. LVB involves lymphatic-venous anastomosis to venules ≤0.8 mm. SOC consists of guideline-based glycemic and risk-factor management, pain control, and standardized wound care. Outcome assessors and statisticians are blinded. The primary outcomes are changes in clinical neuropathy burden and pain severity at 6 and 12 months. Secondary outcomes comprise objective measures of somatic and autonomic physiology, histopathological nerve fiber density, biological serum markers, and longitudinal ulcer epithelialization parameters. Data analysis will use mixed-effects models for repeated measures, with a sample size of 60 adults providing 80% power to detect a conservative between-group effect size of Cohen *d*=0.70.

**Results:**

Recruitment commenced in February 2026 and is planned to continue through July 31, 2027, with follow-up through July 31, 2028. As of May 2026, we have enrolled 3 participants. The first participant has been treated, and a second participant is scheduled to undergo treatment. Data analysis and reporting are anticipated between late 2027 and early 2028. No outcome data are included.

**Conclusions:**

This trial tests a mechanism-based, nonpharmacologic adjunct targeting lymphatic-immune-microvascular dysfunction in DPN. If effective, LVB could inform phenotype-directed treatment algorithms and motivate multicenter evaluation and health economic analyses.

## Introduction

### Background

Diabetic peripheral neuropathy (DPN) is a pervasive and debilitating complication of diabetes that causes significant morbidity and increases the risk of foot ulcers and amputation [1-5]. Its prevalence in Taiwan and neighboring regions is estimated to range from 28.5% to 36.3% [6-8]. Consensus frameworks integrate staged clinical definitions with multimodal diagnostics, including symptom scales (Douleur Neuropathique 4 [DN4], ID-Pain, and Brief Pain Inventory–DPN), bedside sensory testing, autonomic and small-fiber assessments (Sudoscan and electrochemical skin conductance [ESC]), corneal confocal microscopy, quantitative sensory testing (QST), and electrodiagnostics (nerve conduction velocity–electromyography) [9-12]. Despite optimized metabolic care, more than 40% of patients may experience disease progression within 5 years [5]. Analgesics (eg, pregabalin and duloxetine) alleviate painful DPN but do not reverse neuropathy; tricyclic antidepressants require cautious use, and opioids are not first-line therapies [13,14]. Emerging agents such as glucagon-like peptide-1 agonists show preliminary structural neural benefits but require further validation [15]. This creates a critical clinical gap in which existing treatments fail to modify the underlying pathophysiology of the disease, leaving patients at risk of progressive nerve damage and secondary complications.

Beyond fluid homeostasis, the lymphatic system regulates immune trafficking and inflammatory signaling, with lymphatic-cardiovascular cross-talk implicated in metabolic disease and tissue repair [16,17]. Lymphovenous bypass (LVB) (involving ≤0.8 mm anastomoses) is established for lymphedema and has been associated with immunologic rebalancing, reduced oxidative stress, transcriptomic normalization, and improved wound healing [18-23]. The procedure restores drainage and regulates inflammatory signaling, which is hypothesized to reduce endoneurial edema and clear proinflammatory mediators—key drivers of nerve damage in DPN. Our preliminary unpublished case, in which a patient was treated with LVB plus wound care, demonstrated durable healing without amputation and limb-specific neurophysiologic improvement despite suboptimal glycemic control. This clinical observation, combined with the lack of current disease-modifying options, provides a strong rationale for testing LVB as a pathophysiology-targeted intervention to improve small-fiber function and accelerate ulcer healing.

### Aim of the Study

The aim of this study is to evaluate whether LVB combined with standard of care (SOC) provides superior outcomes compared with SOC alone in patients with DPN. We hypothesize that restoration of lymphatic-venous drainage and modulation of inflammatory and oxidative pathways will enhance small-fiber and autonomic function, reduce neuropathic pain, and accelerate ulcer healing when present. To test this hypothesis, the primary outcomes—changes in DN4 and overall neuropathy burden (as measured by the Michigan Neuropathy Screening Instrument [MNSI]) scores—will be evaluated at 6 and 12 months. These outcomes are directly linked to secondary objective measures evaluating somatic and autonomic physiology, including small-fiber sudomotor function (ESC and sympathetic skin response [SSR]), cardiovascular autonomic regulation (heart rate variability [HRV]), thermal and vibratory thresholds (QST), and large-fiber electrodiagnostics (nerve conduction studies [NCSs]). In participants with ulcers, the impact on wound repair will be examined through time to complete epithelialization, healing rates, and complication profiles. Mechanistic insights will be explored through biomarkers of oxidative stress, immune modulation, and intraepidermal nerve fiber density (IENFD), while safety assessments will ensure the tolerability of the intervention.

## Methods

### Design and Setting

This study is a SPIRIT (Standard Protocol Items: Recommendations for Interventional Trials)–compliant, single-center, randomized, controlled, parallel-group superiority trial with a 2:1 allocation ratio (LVB+SOC vs SOC alone). Participants will be stratified based on whether they have an active diabetic foot ulcer—explicitly classified as Wagner Grade ≤2 according to International Working Group on the Diabetic Foot (IWGDF) and Infectious Diseases Society of America (IDSA) criteria—or do not have an active ulcer at the time of enrollment. This uneven allocation maximizes exposure to the novel LVB intervention, prioritizing robust safety and efficacy data for the experimental arm while maintaining a valid and adequately powered control group. Outcome assessors, including Sudoscan and NCS technicians, as well as statisticians will remain blinded to group assignment. The trial will be conducted within the National Taiwan University Hospital system, encompassing both outpatient and inpatient wound care and diabetes clinics.

### Ethical Considerations

The protocol has been approved by the National Taiwan University Hospital Research Ethics Committee (202503048RINA). Prior to any study-related procedures, written informed consent will be obtained from all participants, with an optional component for biospecimen collection. To safeguard privacy and confidentiality, study data will be captured using electronic case report forms in Research Electronic Data Capture (REDCap) with full audit trails, using pseudonymized identifiers to ensure that the data remain deidentified. Continuous safety oversight will be provided by an independent Data and Safety Monitoring Board (DSMB). Our trial has been registered in ClinicalTrials.gov website (NCT07126197). We provide each participant with a convenience store gift card valued at 2000 New Taiwan Dollars (US $64).

### Recruitment and Eligibility

Participant recruitment commenced in February 2026 and is expected to conclude by July 31, 2027, with each participant followed for 12 months. Eligible individuals will be adults aged 20 to 80 years with clinically and electrodiagnostically confirmed DPN, after exclusion of nondiabetic causes, with or without diabetic foot ulcers as defined by the IWGDF and IDSA. Participants must be able to comply with study procedures and provide written informed consent, with optional consent for biospecimen collection. Key exclusion criteria include autoimmune disease or systemic immunosuppression; clinically significant organ failure (cardiac, pulmonary, renal, or hepatic); ankle-brachial index <0.9; baseline infection requiring at least a forefoot amputation; significant cognitive impairment; pregnancy; contraindications to anesthesia or surgery; and known hypersensitivity to blue dyes, including patent blue V or indocyanine green. Recruitment will be conducted through endocrinology, neurology, and wound care clinics, as well as inpatient services. Screening procedures include medical history, DPN staging, validated symptom scales, bedside sensory testing, ESC measurement using Sudoscan, and NCS-electromyography, as indicated. Written informed consent, available in Mandarin and English, will be obtained before initiation of any study-related procedures.

### Randomization, Allocation Concealment, and Blinding

**Figure 1.**
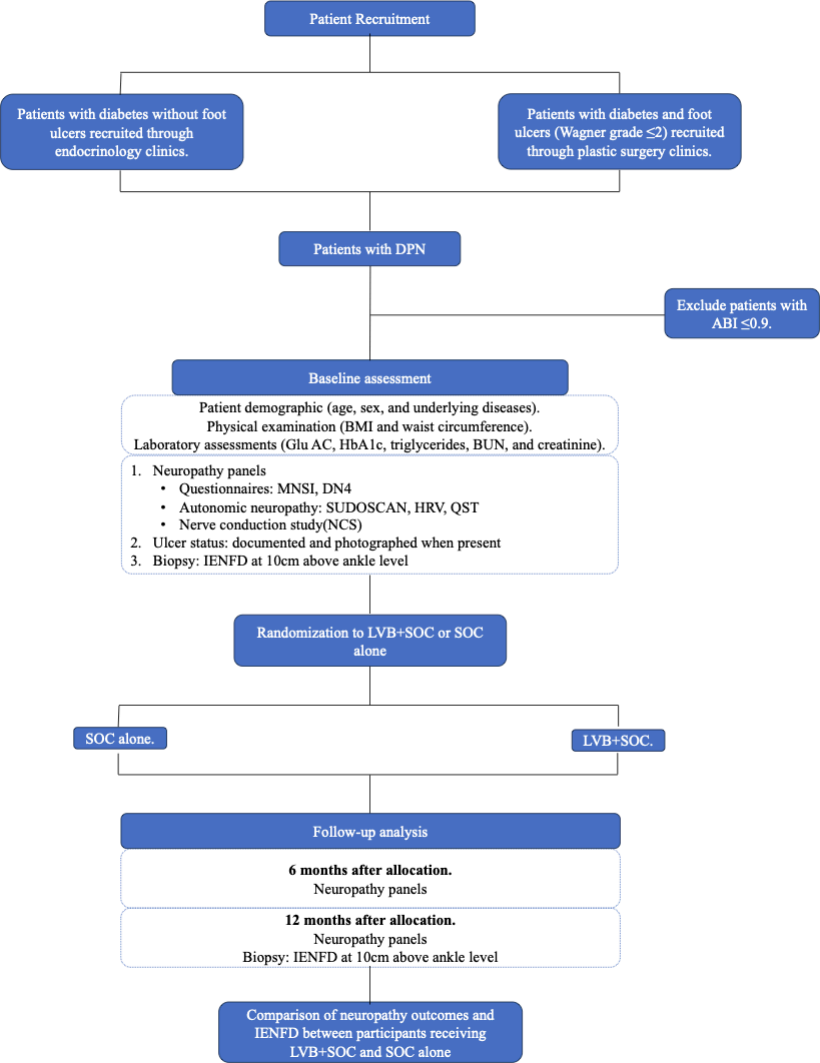
Flowchart of the study design showing participant screening, randomization, allocation to the intervention group (lymphovenous bypass [LVB]+standard of care [SOC]) or the control group (SOC alone), follow-up assessments, and outcome analysis in participants with diabetic peripheral neuropathy (DPN). Outcome assessors and statisticians remain blinded to group allocation. ABI: ankle-brachial index; BUN: blood urea nitrogen; DN4: Douleur Neuropathique 4; Glu AC: glucose *ante cibum*; HbA_1c_: hemoglobin A_1c_; HRV: heart rate variability; IENFD: intraepidermal nerve fiber density; MNSI: Michigan Neuropathy Screening Instrument; QST: quantitative sensory testing.

Participants will be randomized using a computer-generated permuted block design with variable block sizes of 3 and 6. To ensure structural balance, randomization will be stratified within the REDCap module into 2 parallel sequences: stratum 1 (active ulcer present: Wagner grade ≤2) and stratum 2 (no active ulcer). Upon coordinator registration, the participant’s clinical ulcer status will automatically determine the next sequential allocation exclusively from the corresponding stratum block. Neither participants nor surgeons will be blinded due to the nature of the intervention; however, outcome assessors and statisticians will remain blinded to group allocation. To minimize the risk of unblinding, standardized dressings will be used across groups, and independent assessors will conduct outcome evaluations. The enrollment, randomization, intervention, and follow-up processes are summarized in Figure 1 and Table 1.

**Table 1.** SPIRIT (Standard Protocol Items: Recommendations for Interventional Trials)–aligned trial progression, including screening, baseline evaluation, surgical intervention, and longitudinal assessment of study end points.

Trial procedures	Enrollment (month −1)	Baseline (month 0)	Intervention (day 0)	First follow-up (month 6)	Second follow-up (month 12)
Enrollment					
Eligibility screening	✓				
Informed consent	✓				
Randomization		✓			
Interventions					
Lymphovenous bypass+SOC^a^ (experimental)			✓		
SOC alone (control)			✓		
Assessments					
Medical history and BMI		✓			
Laboratory tests (eg, hemoglobin A_1c_)		✓			
*Douleur Neuropathique* 4 questionnaire (neuropathic pain)		✓		✓	✓
Michigan Neuropathy Screening Instrument (diabetic peripheral neuropathy burden)		✓		✓	✓
Sudoscan (electrochemical skin conductance)		✓		✓	✓
Nerve conduction studies–electromyography		✓			
Heart rate variability and quantitative sensory testing		✓		✓	✓
Ulcer photography and ulcer status assessment		✓		✓	✓
Skin biopsy (intraepidermal nerve fiber density)		✓			✓
Biomarkers (oxidative stress)		✓		✓	✓
Adverse event and safety monitoring			✓	✓	✓

a SOC: standard of care.

### Intervention

#### Experimental Intervention (LVB+SOC)

Participants allocated to the experimental arm will undergo LVB in addition to SOC. This SOC consists of guideline-based glycemic and cardiovascular risk-factor management, neuropathic pain control, footwear or offloading, and standardized wound care for participants with ulcers. Routine SOC outpatient visits (eg, routine diabetes and wound clinic appointments at standard intervals) will occur according to standard clinical practice guidelines. Lymphatic mapping will be performed with intradermal blue dye injection, followed by a 2 to 3 cm dorsal foot incision and supermicrosurgical lymphatic-venous anastomosis (end-to-end or end-to-side) to adjacent venules ≤0.8 mm in diameter under microscopy, using 11-0 or 12-0 nylon sutures. At least 1 anastomosis will be completed according to anatomical and flow characteristics, with minimal expected blood loss (<5 mL). In participants with ulcers, concomitant debridement with or without grafting will be undertaken according to IWGDF-IDSA guidelines, followed by standardized postoperative wound care and offloading. Postoperative management will include 24 hours of limb elevation, early mobilization, standard analgesia, and antibiotics if grafting is performed. Treatment fidelity will be maintained by a single credentialed plastic microsurgeon following standardized operating procedures, with intraoperative documentation of the number and type of anastomoses, venous backflow, and confirmation of dye transit.

#### Control Intervention (SOC Alone)

Participants assigned to the control arm will receive SOC only, consisting of guideline-based glycemic and cardiovascular risk-factor management, neuropathic pain control, footwear or offloading, and standardized wound care for those with ulcers. No sham procedure will be performed owing to ethical considerations.

### Outcomes

The primary objective is to compare LVB+SOC with SOC alone in terms of changes in neuropathic pain, assessed by the DN4 questionnaire, and DPN burden, measured by the MNSI at 6 and 12 months. Secondary objectives are to evaluate sudomotor function using Sudoscan, large-fiber nerve conduction parameters, and, in participants with ulcers, time to complete epithelialization adjudicated by blinded review, the proportion of ulcers healed by 20 weeks, infection episodes, reoperations, and rates of minor or major amputation. Exploratory objectives include assessment of serum oxidative stress and antioxidant indices, inflammatory cytokine profiles, and IENFD. The safety objective is to characterize adverse events, serious adverse events, surgical site complications, and hypersensitivity reactions to intraoperative mapping dyes [24].

### Schedule of Assessments

#### Baseline Assessments

At trial inclusion, demographic and clinical variables will be collected, including age, sex, and relevant past medical history (including diabetes mellitus, hypertension, chronic kidney disease, and diabetic retinopathy) from the electronic medical record. Physical examination parameters will include BMI and waist circumference. Laboratory assessments will comprise fasting plasma glucose, glycated hemoglobin (hemoglobin A_1c_), triglycerides, blood urea nitrogen, and serum creatinine. Baseline severity of diabetic neuropathy will be evaluated through NCS, QST, HRV, ESC (measured using Sudoscan, if not performed within the preceding 3 months), and symptom scoring with the MNSI and DN4. Ulcer status will be documented and photographed when present, and IENFD from ulcer biopsy specimens will be evaluated by a neurologist.

#### Follow-Up Assessments

At 6 months, participants will undergo repeat assessments, including Sudoscan, HRV, QST, MNSI, and DN4, with testing supported by self-payment arrangements. Additionally, any changes in time-varying covariates, such as concomitant treatments (eg, adjustments to neuropathic pain medications or glycemic control agents), will be systematically reviewed and recorded. At 12 months, similar assessments (Sudoscan, HRV, QST, MNSI, and DN4) and IENFD evaluation will be performed, with reimbursement provided under the National Health Insurance framework. These follow-up evaluations are intended to capture both the primary end point at 6 months and the durability of outcomes at 12 months.

### Statistical Analysis

A total sample size of 60 randomized participants (LVB n=40; SOC n=20) provides 80% power (*α*=0.05) to detect a conservative between-group effect size of Cohen *d*=0.70 for the primary end point. This target randomized sample size accounts for an anticipated 15% dropout and missing data rate across both groups to ensure that the study remains adequately powered for the final analysis. This calculation was performed using G*Power (version 3.1.9.7) based on a 2-sample *t* test framework to ensure robust power for the subsequent longitudinal analysis. All statistical analyses will be performed using SPSS (version 28.0; IBM Corp). The primary analysis will follow the intention-to-treat principle, with per-protocol analyses conducted as sensitivity checks. The primary end point—jointly defined as the changes in DN4 and MNSI at 6 and 12 months—will be analyzed using a mixed-effects model for repeated measures (MMRM). Continuous secondary end points, including ESC values, SSR latencies or amplitudes, HRV parameters, and QST thresholds, will be analyzed using identical, parallel MMRM frameworks to control for baseline values and time-varying covariates. Exploratory biomarker analyses will use analysis of covariance or mixed models, with multiple testing controlled by the Benjamini-Hochberg false discovery rate. Missing data will be assumed to be missing at random, with MMRM inherently accommodating missingness and multiple imputation using Multiple Imputation by Chained Equations applied for sensitivity analyses. To ensure stability, given the small control group (n=20), Multiple Imputation by Chained Equations will use a restricted set of covariates. An anticipated dropout and missing data rate of 15% is expected across both groups. Prespecified exploratory, hypothesis-generating subgroup analyses will evaluate outcomes by ulcer status, baseline DPN stage, hemoglobin A_1c_ tertiles, dyslipidemia, and chronic kidney disease. Interim analyses will be restricted to safety reviews by the DSMB after approximately 20 and 40 participants, with no efficacy-based stopping rules.

### Data Management

The study data recorded using electronic case report forms will be retained for at least 5 to 10 years. Risk-based monitoring will be performed, with 100% source data verification for the primary end point and informed consent. LVB is considered a low-risk microsurgical procedure, and participant safety will be overseen by an independent DSMB.

## Results

This study is currently in the active recruitment and intervention phase, having officially commenced enrollment in February 2026. We have successfully enrolled 3 participants, and the first experimental case of LVB has been completed. Preliminary clinical follow-up for the first participant has been encouraging, with the participant reporting increased plantar sensation postoperatively; objective Sudoscan follow-up assessments are pending. A second participant is scheduled to undergo the surgical intervention in May 2026. Each participant will be followed for 12 months, with final follow-up expected by July 31, 2027. Data lock and analysis are planned for the third and fourth quarters of 2027, and primary reporting of study outcomes is anticipated between the fourth quarter of 2027 and the first quarter of 2028. No outcome data are available at this time.

## Discussion

This trial evaluates LVB as a mechanism-based adjunctive therapy for DPN. Regarding our principal findings, we anticipate that supermicrosurgical restoration of lymphatic-venous drainage will significantly improve small-fiber and autonomic function—reduce neuropathic pain and increase ESC—and accelerate epithelialization in participants with active ulcers. In comparison to prior work, this localized surgical approach addresses a critical gap left by conventional pharmacotherapies that merely treat symptoms without modifying the underlying pathophysiology. This protocol is the first clinical trial evaluating LVB specifically for DPN, paralleling successes observed in populations with lymphedema and venous ulcers. The trial’s major strengths include its randomized, assessor-blinded design and the integration of biomarker and histological substudies. Finally, our dissemination plan ensures that these findings will be shared through peer-reviewed medical literature and international congresses to inform future precision care algorithms.

Reduced IENFD is a critical histopathological marker of diabetic neuropathy that significantly contributes to the pathogenesis and impaired healing of diabetic foot ulcers. The progressive loss of these small nerve fibers, driven by metabolic and microvascular injury, leads to diminished protective sensation and disruption of neurotrophic and neurovascular support. This lack of sensory feedback allows minor foot injuries to go unnoticed, while the absence of key neuropeptides, such as calcitonin gene-related peptide and substance P, impairs local blood flow and tissue regeneration. The combination of compromised sensation and a blunted healing response creates a vicious cycle that contributes to the chronicity and high morbidity of these ulcers [25,26]. Therefore, the assessment of IENFD, often through skin biopsies, serves as a valuable diagnostic and prognostic tool by offering insight into the neural contributions to ulcer pathogenesis and serving as a sensitive end point for evaluating therapeutic efficacy.

Diabetic autonomic neuropathy is a key factor in the development and poor healing of diabetic foot ulcers. Assessments such as HRV and SSR are essential for evaluating this condition. A decrease in HRV indicates parasympathetic dysfunction, increasing cardiovascular risk, while an absent or reduced SSR indicates sympathetic nerve failure, causing dry, cracked skin. This dual impairment—loss of protective sensation from somatic neuropathy and compromised skin integrity from autonomic dysfunction—creates a cycle in which minor injuries go unnoticed, and the body’s natural healing processes are hindered, leading to chronic, nonhealing ulcers [27,28]. Therefore, a comprehensive assessment of both somatic and autonomic neuropathy is vital for effective management and risk stratification in patients with diabetic foot ulcers. We will evaluate the effectiveness of LVB in alleviating diabetic autonomic neuropathy.

The strengths of this study include its randomized, assessor-blinded design, the incorporation of objective autonomic and electrophysiologic end points, and the integration of biomarker and histological substudies to provide mechanistic insight. The inclusion of ulcer-related outcomes further enhances the clinical relevance of the study, as foot ulcers remain a major cause of morbidity and amputation in DPN. Positive findings, particularly improvements in small-fiber function and ulcer healing, could inform future treatment algorithms tailored to specific DPN phenotypes, thereby advancing precision care in diabetic neuropathy.

Several limitations must be acknowledged. The single-center design may constrain generalizability, and the inability to blind participants and surgeons introduces a risk of performance bias, although this limitation is mitigated by the use of objective, assessor-blinded measures such as ESC and NCS. The heterogeneity of DPN phenotypes, including the broad age range (20-80 years) included in our eligibility criteria, presents additional challenges in interpreting subgroup responses; however, our statistical model adjusts for age and diabetes duration to mitigate this issue. Although the sample size is adequately powered for the primary end point, it may be underpowered to detect smaller effects across secondary and exploratory outcomes. The small sample size, particularly in the SOC control group (n=20), poses a significant constraint relative to the planned complex multivariable MMRM analyses and increases the risk of unstable estimates. Future research should include multicenter replication to validate findings, cost-effectiveness analyses to assess feasibility in routine practice, and refinement of indications such as early autonomic DPN or refractory ulcer phenotypes. Comparative effectiveness studies, including evaluation alongside emerging pharmacologic agents such as glucagon-like peptide-1 receptor agonists, are also warranted. Taken together, this trial has the potential to establish LVB as a paradigm-shifting intervention that redefines the therapeutic landscape of DPN.

## Abbreviations

DN4: Douleur Neuropathique 4
DPN: diabetic peripheral neuropathy
DSMB: Data and Safety Monitoring Board
ESC: electrochemical skin conductance
HRV: heart rate variability
IDSA: Infectious Diseases Society of America
IENFD: intraepidermal nerve fiber density
IWGDF: International Working Group on the Diabetic Foot
LVB: lymphovenous bypass
MMRM: mixed-effects model for repeated measures
MNSI: Michigan Neuropathy Screening Instrument
NCS: nerve conduction study
QST: quantitative sensory testing
REDCap: Research Electronic Data Capture
SOC: standard of care
SPIRIT: Standard Protocol Items: Recommendations for Interventional Trials
SSR: sympathetic skin response
